# Natural foods resources and dietary ingredients for the amelioration of *Helicobacter pylori* infection

**DOI:** 10.3389/fmed.2023.1324473

**Published:** 2023-12-06

**Authors:** Chengyuan Wang, Meixiang Yao, Hongguang Zhong, Stephene S. Meena, Fuxing Shu, Shaoping Nie, Mingyong Xie

**Affiliations:** ^1^State Key Laboratory of Food Science and Technology, China-Canada Joint Laboratory of Food Science and Technology (Nanchang), Key Laboratory of Bioactive Polysaccharides of Jiangxi Province, Nanchang University, Nanchang, Jiangxi, China; ^2^Jiangzhong Dietary Therapy Technology Co. Ltd, Jiujiang, Jiangxi, China; ^3^Jiangzhong Cancer Research, Jiangxi University of Chinese Medicine, Nanchang, Jiangxi, China; ^4^College of Biotechnology and Pharmaceutical Engineering, Nanjing Tech University, Nanjing, Jiangsu, China

**Keywords:** *Helicobacter pylori*, antibiotics, natural foods, eradication, dietatary ingredients

## Abstract

*Helicobacter pylori (H. pylori)* is a gastric-persistent pathogen that can cause peptic ulcer disease, gastric cancer, and mucosal-associated lymphoid tissue lymphoma. This pathogen is commonly treated with antibiotic-based triple or quadruple therapy. However, antibiotic therapy could result in the bacterial resistance, imbalance of gut microbiota, and damage to the liver and kidneys, etc. Therefore, there is an urgent need for alternative therapeutic strategies. Interestingly, natural food resources, like vegetables, fruits, spices, and edible herbs, have potent inhibitory effects on *H. pylori*. In this review, we systematically summarized these foods with supporting evidence from both animal and clinical studies. The results have indicated that natural foods may possess temporary inhibition effect on *H. pylori* rather than durable eradication, and may help to reduce *H. pylori* colonization, enhance the effect of antibiotics and modulate the host’s immune response.

## Introduction

**Figure 1:**
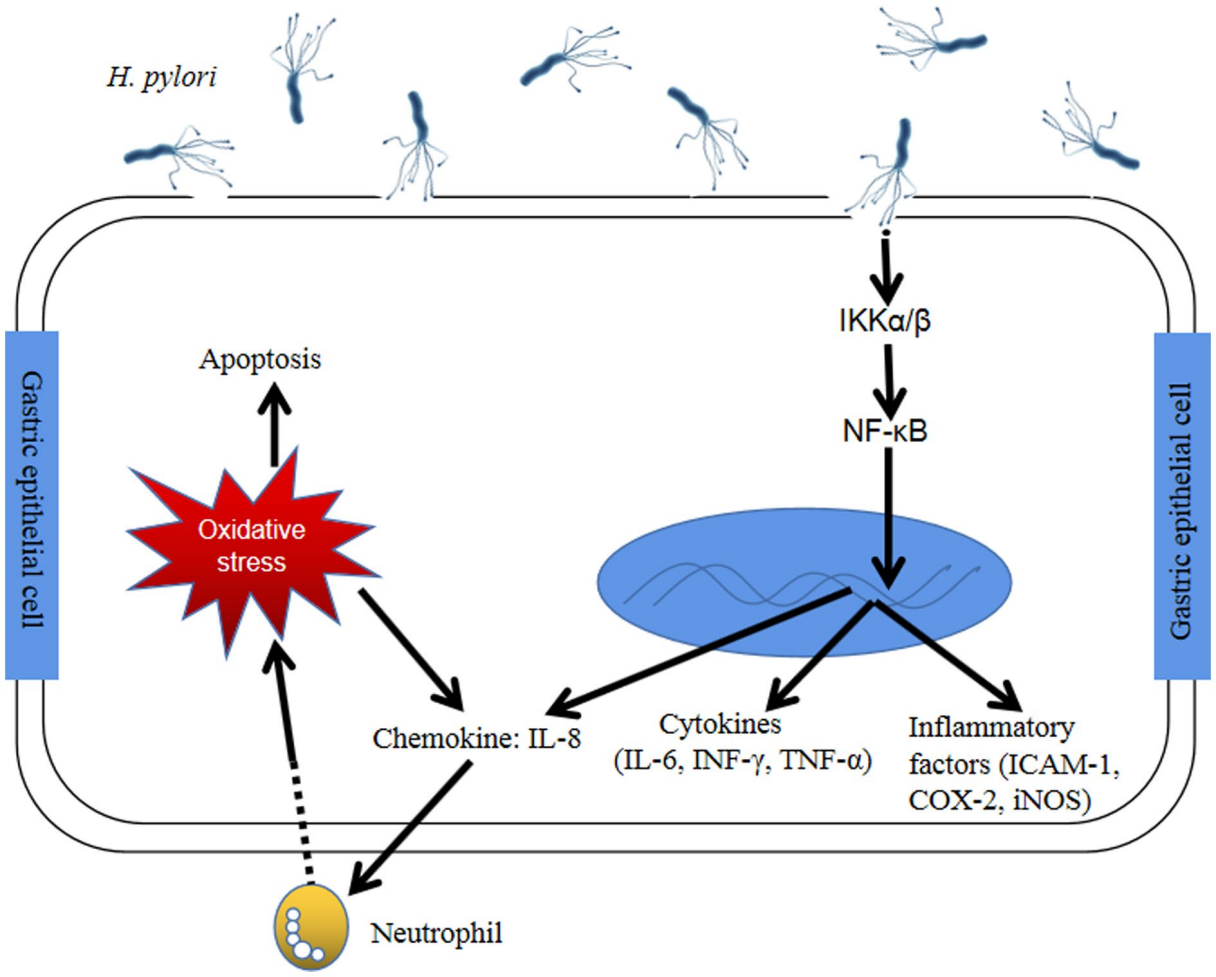
The immune response of gastric epithelial cells after *Helicobacter pylori* infection through NF-κB pathway (2) *H. pylori* infection activates NF-κB-related kinase, like IκB α/β, resulting in IκBα of NF-κB phosphorylation and degradation. Active NF-κB translocates into the nucleus and trigger the translation of inflammatory cytokines (COX-2, ICAM-1 and iNOS) and proinflammatory factors (NF-κB, IL-1, −6, −8, TNF-α). Furthermore, *H. pylori* could cause the generation of intracellular reactive oxygen species by host gastric epithelial cells and eventually led to cell apoptosis. Abbreviation: NF-κB: Nuclear factor-kappaB; IKKα/β: IκB kinase α/β; INF-γ: Interferon-γ; TNF-α: Tumor necrosis factor-α; IL: Interleukin; ICAM-1: Intracellular adhesion molecule-1; COX-2: Cyclooxygenase-2; iNOS: Inducible nitric oxide synthase.

*Helicobacter pylori (H. pylori)* is a gram-negative, microaerophilic spiral-shaped bacterium, which is classified as a Group I carcinogen by the World Health Organization. It can persistently inhabit in the stomach, which can lead to chronic gastritis, gastric ulcer, gastric cancer, and mucosa-associated lymphoid tissue lymphoma (MALT). About 70–80% of the world’s population in developing countries are infected with *H. pylori*, whereas,in developed countries, the infection rate is 13–50% (1). After infection and colonization with *H. pylori*, gastric epithelial cells trigger a series of immune response at both the celluar and molecular levels, such as NF-κB pathway (Figure 1), eventually causing gastric diseases (3). Patients infected with *H. pylori* are at higher risk for gastric ulcer and cancers (2). The infection of *H. pylori* may increase the risk of developing gastric MALT about 50 to 73% (4). In about 1% of gastric cancer patients infected with *H. pylori*, there co-exists increasing inflammation of the gastric corpus, the atrophic mucous membrane in the prepyloric stomach, and reduced gastric acid secretion (5). Furthermore, *H. pylori* infection has also been reported to be associated with insulin resistance, nonalcoholic steatohepatitis, type 2 diabetes mellitus, etc. (6). The main routes of *H. pylori* transmission are mouth-to-mouth, fecal-to-mouth and spread between family members (7).

Until now, *H. pylori* eradication in infected individuals remains a good choice and the most direct approach to prevent the development of *H. pylori*-related gastric disorders and gastric cancer (8). In general, first-line treatment for eradication of *H. pylori* consists of standard triple therapy (consisting of a proton pump inhibitor (PPI) and two of three antibiotics: clarithromycin and either amoxicillin or metronidazole) and bismuth-based quadruple therapy (consisting of bismuth with PPI and two antibiotics) (9, 10). The estimated efficacy of triple therapy is 82% and that of sequential therapy is 92% (11). However, various studies have reported that there are two limiting factors impairing the eradication of *H. pylori*, which involve development of bacterial resistance to antibiotics and the persistence of low levels of *H. pylori* bacteria in gastric epithelial cells (12). As we known, standard antibiotic therapy is high cost and requires at least fourteen days of drug administration and may be associated with some side effects such as diarrhea, nausea, and taste disturbances, leading to poor patient compliance (11). In addition, there are significant concerns about the issue of bacterial resistance, unaffordable treatment expenses, treatment tolerability, and cultural acceptability of antibiotic treatment (13). Apparently, long-term antibiotic therapy could lead to alarming imbalances of microbiota in the gut, promoting the growth of resistant strains of *H. pylori* or the emerging of other harmful micro-organisms such as *Candida fungi* and *Clostridium difficile* (14).

However, *H. pylori* has co-ecolved with humans for a long time. The human body is a complex or multiorganism ecosystem in which any chang may effect human health (15, 16). The interactions between microbiota and host are complicated and poorly understood (17). There are a few reports in literature suggesting that human beings benefit from *H. pylori* (10). For instance, *H. pylori* could protect children against gastrointestinal infection (18), reducing the prevalence of atopic diseases such as celiac disease, irritable bowel syndrome (IBS) and gastroesophageal reflux disease (GORD), etc. (19). Therefore, research into alternative or novel strategies to prevent and treat *H. pylori* infection has recently attracted the attention of scientists.

Notably, many natural food resources have been consumed by people since ancient times to treat gastrointestinal diseases (20) and anti-cancer (21), through which recent studies have been proven to be effective and have few side effects. These natural resources are an invaluable treasure for developing or mining drugs to treat human diseases (22). Numerous studies recently have shown that natural food resources including vegetables, fruits, spices and edible herbs contain powerful and valuable anti-*H. pylori* activities (12).

Herein, we have systematically reviewed published literature with supporting evidence of animal and clinical studies from the PubMed, ClinicalTrials.gov and Scopus databases. The following terms of *Helicobacter pylori*, food, vegetable, fruit, spices, plants, and / or herbs in all possible combination have been used for retrieval.

**Figure 2:**
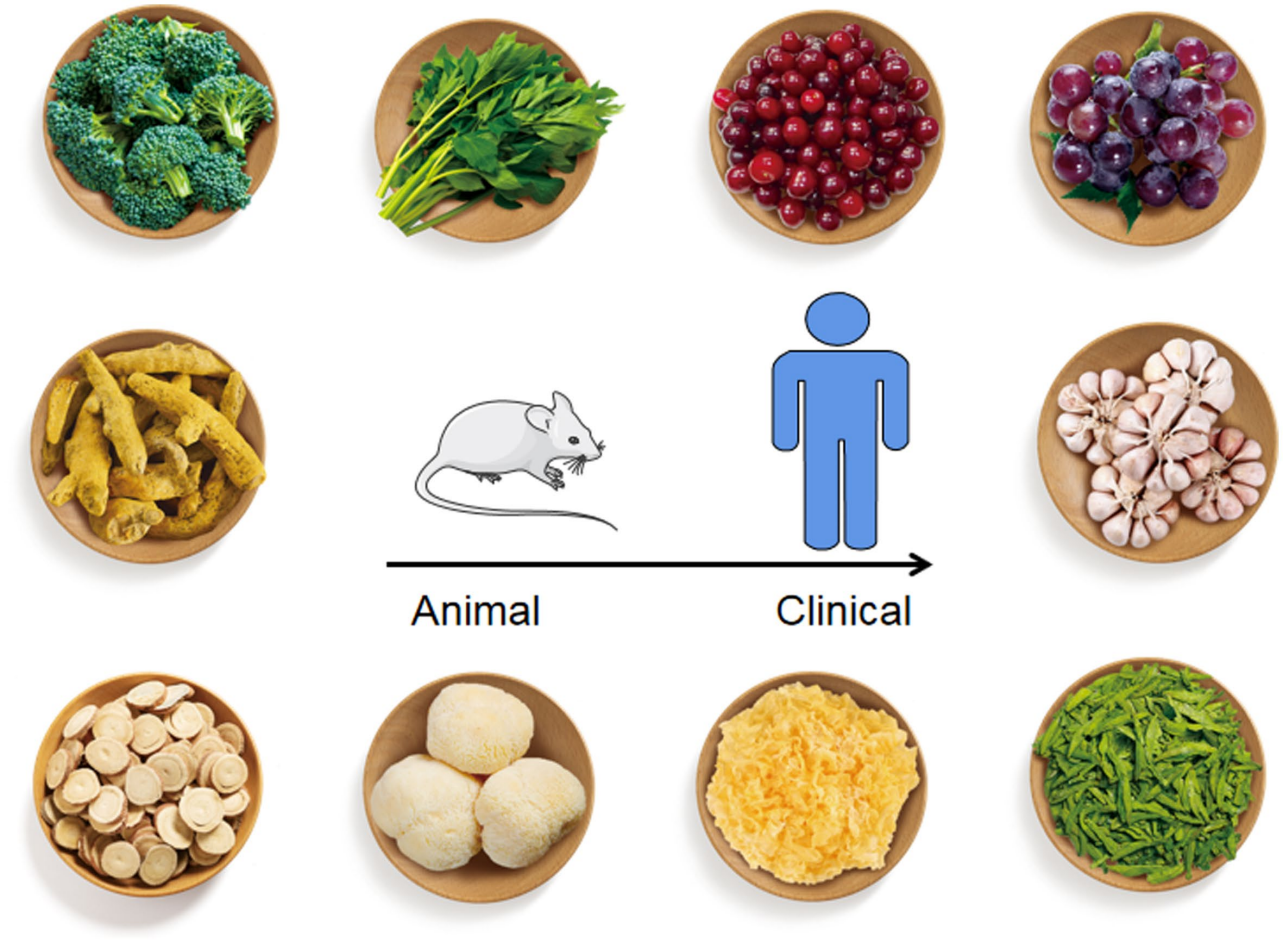
Natural foods listed in this review with anti-*H. pylori* effect. From top to bottom, left to right, the foods figures referred to broccoli, *Angelica keiskei*, cranberry, grape, curcumin, garlic, liquorice, *Hericium erinaceus*, *Tremella mesenterica*, and green tea.

We expected sincerely that the appropriate combination of dietary ingredients from natural foods could be utilized to prevent, manage or treat *H. pylori* infection or / and related diseases such as gastric cancer. In this review, we have summarized the animal and clinical studies on the use of these natural food resources to relieve *H. pylori*-related infection (Figure 2).

**Figure 3:**
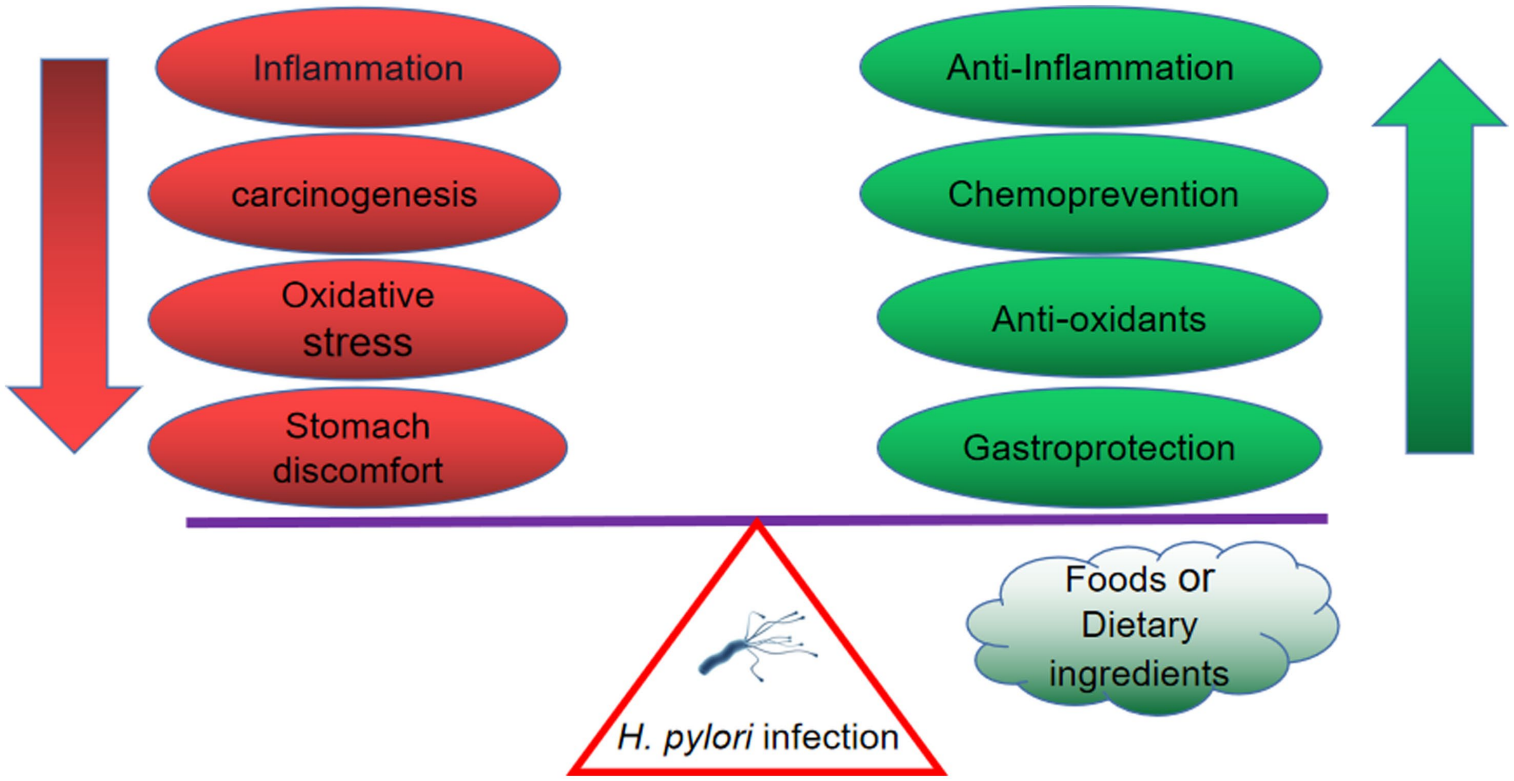
The *H. pylori*-infected individual benefits from natural products, including anti-inflammation, anti-oxidative, chemoprevention and gastroprotection.

In addition, a majority of individuals with *H. pylori* infection remain asymptomatic, but they are still at risk of developing the illness associated with this bacterium. However, the eradication of *H. pylori* in the case of asymptomatic patients in developing countries is not practical. Alternative therapies have proved to be useful in anti-inflammation, anti-oxidative, chemoprevention and gastroprotection. Based on these results, the inclusion of foods from natural products and dietary ingredients in the diet of asymptomatic patients could reduce the risk and development of *H. pylori* infection. Herein, the benefits from these alternative therapies are summarized in Figure 3. The summary of vegetables, fruits, spices, and edible herbs possessing anti-*H. pylori* activities is presented below.

### Suppressive effects of vegetables on *Helicobacter pylori* infection *in vivo*

Study showed that people with a lower dietary intake of vegetables were at higher risk of *H. pylori* infection (23). Recently, the effects of various vegetables on *H. pylori* infectionwere studied. For instance, broccoli is a common vegetable that can be consumed as food and for medicinal purposes, it showed that broccoli has both anti-cancer and anti-bacterial activity (24). Broccoli sprouts, in particular, are rich in isothiocyanate sulforaphane (SF) in the form of a precursor called glucoraphanin, which has shown potent bacteriostatic activity against *H. pylori* (12). Lozniewski and colleagues revealed that *H. pylori* infections could be eradicated in 8/11 transplants treated with 7.5 μmol sulforaphane by using human gastric xenografts model in nude mice, which also suggested individuals with *H. pylori* infection might benefit from sulforaphane (25).

Similarly, Yanaka et al. (26) investigated the protective effect of broccoli sprouts against high salt-induced gastritis in a mouse model of *H. pylori* infection. The results showed that the sulforaphane contained in broccoli sprouts was effective in inhibiting colonization, inflammation and gastric mucosal atrophy. Furthermore, the asymptomatic patients with *H. pylori* infection consumed broccoli sprouts (70 g/d containing 420 μmol glucoraphanin), resulted in a significant decrease in *H. pylori* colonization and gastric inflammation, but these effects disappeared 2 months later without following intake (26). Meanwhile, a preliminary study reported temporary eradication of *H. pylori* in four out of nine *H. pylori* infected subjects who were treated with broccoli sprouts (27). Broccoli sprouts (6 g/d sulforaphane-rich) plus triple therapy treatment could significantly decrease infected patients’ systolic and diastolic blood pressure in 86 patients with type 2 diabetes (28). *H. pylori* density in infected patients was not significantly improved by treatment with broccoli sprouts, but it reduced malondialdehyde (MDA) values, indicating that broccoli sprout could inhibit the lipid peroxidation and exert cytoprotection effects on *H. pylori* related gastritis (29).

In addition, a leafy green vegetable, *Angelica keiskei*, was found to exert inhibitory effects on *H. pylori*-induced gastric inflammation in mice, and the possible mechanism of action could be based on the inhibition of inflammatory mediators (IFN-γ, COX-2, and iNOS) mediated by NF-κB signaling pathways (30). Another vegetable, *Nigella sativa*, have many active ingredients with potent medical effects such as antimicrobial, anti-inflammatory, and anti-cancer activities (31). The result of *N. sativa* seeds against *H. pylori* infection in 88 patients with nonulcer dyspepsia showed that, the eradication rates achieved by triple therapy, 1 g, 2 g, and 3 g ground *N. sativa* seeds were 82.6, 47.6, 66.7, and 47.8%, respectively. Notely, there was no statistically significant difference of the eradication rate between triple therapy and 2 g of seeds powder (32).

Above all, it is evident that dietary ingredients from vegetables such as broccoli sprout, *A. keiskei* and *N. sativa* have effects on inhibition of *H. pylori* colonization, enhancement of antibiotic sensitiveness and suppression of *H. pylori*-induced inflammation or oxidative stress *in vivo*. All results are sorted out in Table 1.

**Table 1:** Suppressive effects of vegetables on *Helicobacter pylori* infection *in vivo.*

Vegetables	Components	*In vivo* models	Study sample	Main results	References
Broccoli sprout	7.5 μmol sulforaphane	Nude mice	Two groups: treated group (7.5 μmol sulforaphane per day) and control group (without sulforaphane)	H *pylori* was completely eradicated in 8 of the 11 sulforaphane-treated grafts	(25)
Broccoli sprout	3 μmol of glucoraphanin	C57BL/6 mice	Two groups: treated group (administration of homogenized broccoli sprouts) and untreated group	Inhibition of colonization, inflammation, and gastric mucosal atrophy of *H. pylori*-infected mouse	(26)
Broccoli sprout	420 μmol of glucoraphanin	*H. pylori* positive human	Two groups: broccoli sprout group (*n* = 25) and alfalfa group (*n* = 23)	Significant reduction in the levels of urease and serum pepsinogens I and II.	(26)
Broccoli sprout	7, 14 or 28 g broccoli sprouts	Human with *H. pylori* infection	Nine *H. pylori*-infected subjects	Eradication of *H. pylori* colonization following broccoli sprout treatment in 4/9 subjects	(27)
Broccoli sprout	Broccoli sprouts powder (6 g per day) alone or in combination with triple therapy	*H. pylori* positive human with type 2 diabetes patients	86 type 2 diabetes patients with positive *H. pylori* stool antigen test (77 patients completed trial) were assigned three groups: A: broccoli sprouts power; B: standard triple therapy; C: broccoli sprouts power + standard triple therapy	Eradication rates were 56 and 91.7% with broccoli sprouts power alone or in combination with triple therapy. In addition, broccoli sprouts powder improved cardiovascular risk factors.	(28)
Broccoli sprout	250 mg broccoli sprout extract containing 1 mg sulforaphane	*H. pylori* positive or placebo subjects	Three groups: group A (Hp^+^, broccoli sprout extract containing sulforaphane, *n* = 33); group B (placebo, *n* = 28); group C (Hp^−^, broccoli sprout extract containing sulforaphane, *n* = 28)	No significant improvement on *H. pylori* density in group A. Significantly reduced the malondialdehyde (MDA) values in group A and C.	(29)
*Angelica keiskei*	*A. keiskei*	C57BL/6 mice	Four groups (12 mice per group): None (animals without *H. pylori*); *H. pylori* control (animals with *H. pylori* infection); *H. pylori* + *A. keiskei*; *H. pylori* + N-acetylcysteine	Increase of *H. pylori*-induced lipid peroxide and the enhancement of myeloperoxidase activity, suppressed the expression of INF-γ, COX-2, and iNOS, and inhibited gastric neutrophils infiltration. Also, it blocked NF-κB activation, and maintained the IκBα protein at a higher level in *H.pylori* infected mice.	(30)
*Nigella sativa*,	The powder of its seeds	*H.pylori* infected patients with nonulcer dyspepsia	88 patients were administered with triple therapy, 1 g, 2 g, and 3 g of ground *N. sativa* seeds, respectively.	The eradication rates achieved by triple therapy, 1 g, 2 g, and 3 g ground *N. sativa* seeds were 82.6, 47.6, 66.7, and 47.8%, respectively.	(32)

### Suppressive effects of fruits on *Helicobacter pylori* infection *in vivo*

Epidemiological studies have shown that the risk of gastric cancer was lower in people with more fruit consumption (33). Fruits raw extract or active ingredients had inhibition effects on the growth of *H. pylori* growth (13), and anti-cancer (34). Based on the current notion that *H. pylori* is a key factor in the development of gastric cancer, it is easily deduced that inhibition of *H. pylori* activity may play an important role in reducing gastric cancer risk.

Among numerous fruits of the berries, such as cranberry, bilberry, raspberry, elderberry and strawberry, it has been widely focused their effect on inhibiting *H. pylori* and enhancing antibiotics sensitive to *H. pylori* (40). Cranberries are a great source of dietary ingredients, such as anthocyanins and proanthocyanins with high value of health benefits (41). There are a few studies that have examined the efficacy of cranberry juice on mice or human infected with *H. pylori*.

Cranberry juice could eliminate 80% of *H. pylori* colonization in infected mice after 24 h of intervention, however, the eradication rate reached only 20% after 4 weeks of treatment (35). A double-blind, randomized, placebo-controlled study of regular consumption of cranberry juice in infected Chinese subjects had yielded significantly negative ^13^C-urea breath test results (14/97 in the treatment group versus 5/92 in the placebo group) after 90 days intervention (36). The other study showed that the negative rate of *H. pylori* in female and male patients, who were administered with triple therapy during the first week and followed by cranberry supplements for two weeks, was 95.2 and 73.9%. The results indicated that the female patients may obtain more benefit form the addition of cranberry (37). Gotteland et al. (38) had shown that the *H. pylori* eradication rate reached 16.9% in asymptomatic infected children by daily intake of cranberry juice for three weeks, although the clearance effect disappears after cessation of cranberry juice consumption.

Grape may be the other fruit that has reported the positive effects on human infected with *H. pylori*. Brown et al. (39) tested the activity of grape skin together with quercetin did not significantly inhibit the growth of *H. pylori,* but was effective in reducing inflammatory cytokines including TNF-α, IL-1 and IFN-γ.

From the results mentioned above and summarized in Table 2, it is clear that fruits, especially like cranberry, could suppress the growth of *H. pylori,* and help infected subjects to attenuate the levels of inflammatory factors. The inhibitory effects of fruits may be sourced from polyphenols (34). Cranberry components showed synergistic effects with antibiotics. The potential mechanisms might be that components firstly damage the cell membranes of *H. pylori*, and then make cells more sensitive to antibiotics (34).

**Table 2:** Suppressive effects of fruits on *Helicobacter pylori* infection *in vivo.*

Fruits	Components	*In vivo* models	Study sample	Main results	References
Cranberry	Cranberry juice cocktail	C57BL/6 mice	80 infected mice were randomly allocated into four groups (20 mice in each group): Control group (untreated); group A (cranberry juice); group B (triple therapy: amoxycillin, bismuth subcitrate and metronidazole); group C (cranberry juice + triple therapy)	The eradication rates in group A, B and C were 20, 80 and 80% compared with the control group.	(35)
Cranberry	Cranberry juice cocktail	189 adults aged 48.9 ± 11.2 years	Volenteers were randomly divided into two groups: cranberry juice treatment group (*n* = 97); placebo group (*n* = 92).	At day 35 or 90 of intervention, 14 of the 97 from the cranberry juice treatment group and 5 of the 92 of the placebo group had negative ^13^C-urea breath test results.	(36)
Cranberry	Cranberry juice	177 patients with *H. pylori* infection	The patients were randomly allocated to receive 250 mL of either cranberry juice (cranberry-OAC, *n* = 89) or placebo beverage (placebo-OAC, *n* = 88) twice daily and only cranberry juice or placebo beverage for the next 2 weeks.	These results suggest that the addition of cranberry to triple therapy improves the rate of *H. pylori* eradication in females.	(37)
Cranberry	Cranberry juice	295 asymptomatic children colonized with *H. pylori*	A multicentric, randomized, controlled, double-blind trial was carried out. All subjects were allocated four groups: control group (placebo juice/heat-killed La1); CB group (cranberry juice/heat-killed La1); CB/La1 group (Cranberry juice/La1); La1 group (placebo juice/La1).	The *H. pylori* eradication rates increase significantly in La1(14.9%), CB(16.9%), and CB/La1(22.9%).	(38)
Grape	Skin and quercetin	Mice	Mice were treated with 5 and 10% grape skin or quercetin (25 mg kg^−1^ body weight)	Grape skin and quercetin effectively reduced inflammatory cytokines (TNF-α, IL-1β and IFN-γ)	(39)

### Suppressive effects of spices on *Helicobacter pylori* infection *in vivo*

Spices have been widely used as food flavors and therapeutic ingredients since ancient times. Until now, spices have been reported to process anti-cancer effects, particularly in gastric and colon cancer (42). *H. pylori* is one of the risk factors for gastric cancer, the anti-*H. pylori* effect of spices may play a crucial role in preventing the development of gastric cancer. Moreover, Liu et al. (34) have concluded that spices have anti*-H. pylori* activity.

Curcumin is a polyphenolic yellow pigment which is rich in turmeric root. Turmeric is widely used as spices, food coloring and medicines in India and Southeast Asia (43). Curcumin presented strong therapeutic effect against *H. pylori* infection for it not only could eradicate *H. pylori,* but also completely repair the gastric damage induced by *H. pylori* (44). 200 or 600 mg/kg of curcumin had anti-inflammatory effect by suppressing expression of NF-κB p65 (45). Similarly, Kundu et al. (46) revealed that curcumin had healing-promoting effect on *H. pylori* infected mice and could restrict the expression of metalloproteinases-3 and 9 (46).

Furthermore, clinical studies have been conducted for exploring the inhibition effects of curcumin on *H. pylori.* Judaki et al. (47) investigated the clinical effect of curcumin (700 mg / three times a day for 28 days) combined with triple therapy regimens for patients with chronic gastritis-associated *H. pylori* infection. The result suggested that the additive curcumin could significantly decrease MDA level and glutathione peroxides, improve the total antioxidant capacity of the gastric mucosa, increase the *H. pylori* eradication rate compared to triple therapy alone (86.4% vs. 74.5%, *p* < 0.05) (47).

The other clinical study showed that standard triple therapy with curcumin significantly improves dyspeptic symptoms, despite no obvious effect on *H. pylori* eradication (48). Similar results have been reported by di Mario et al. (49), they found that curcumin-based therapy could relieve functional dyspeptic symptoms as well as serological signs of gastric inflammation, and increase 12% level of eradication rate (49). In another case, the *H. pylori* eradication rate was only 5.9% when patients were treated with curcumin alone, however, extra finding was that curcumin could modulate the production of inflammatory cytokines (50).

Garlic (*Allium sativum*), the other food flavoring, shares high scores in therapeutic properties (51). It has been reported that garlic consumption has therapeutic benefits in precancerous gastric lesions (52). Limuro et al. showed that the water-ethanol extract of garlic was able to inhibit *H. pylori*-induced early gastritis in a dose-dependent manner in Mongolian gerbils (53). Moreover, the administration of 4 g/day garlic powder increased the eradication rate of *H. pylori* in the treatment group (87%) compared to the placebo group (73%), although the difference was not statistically significant (54).

In general, these results pointed toward that spices have potent healing effect on gastric damage caused by *H. pylori* (Table 3). All above observations not only suggest the therapeutic effect of curcumin against *H.pylori* infections but underline the anti-inflamatory effect of curcumin. Curcumin may be as a potential therapeutic candidate for *H.pylori* associated disease.

**Table 3:** Suppressive effects of spices on *Helicobacter pylori* infection *in vivo.*

Spices	Components	*In vivo* models	Study sample	Main results	References
Curcumin	25 mg/kg body weight (once daily for 7 d)	C57BL/6 mice	Treatment mice group were was orally fed with curcumin 25 mg/kg once daily for 7 d; Untreated mice group only received sterile water.	Curcumin was highly effective in two aspects: eradication of *H. pylori* and restoration of gastric damage induced by *H. pylori*.	(44)
Curcumin	200 or 600 mg/kg body weight curcumin	Sprague–Dawley rats	25 male SD rats were equally divided into five groups: 1, control rats. 2, control rats +600 mg/kg curcumin. 3, *H. pylori* infected rats. 4, *H. pylori* infected rats +200 mg/kg curcumin. 5, *H. pylori* infected rats +600 mg/kg curcumin.	Curcumin reduces the *H. pylori-* induced gastric inflammation in rats’ model. Curcumin could deduce macromolecular leakage through the suppression of NF-κB expression in gastric epithelium cells.	(45)
Curcumin	curcumin (25 mg/kg or 50 mg/kg b.w.) + triple therapy	C57BL/6 mice	The mice were orally fed with curcumin (25 mg/kg or 50 mg/kg b.w.) or triple therapy (Omeprazole, tinidazole and amoxicillin) or only-antibiotics (tinidazole and amoxicillin), for 7 days, while untreated ones received sterile water and curcumin control group received only curcumin.	Curcumin is capable of eradicating *H. pylori*-infection in mice. The mechanism by which curcumin protects *H. pylori* infection may involve the regulation of metalloproteinase 3 and 9 expression.	(46)
Curcumin	Standard triple therapy (omeprazole, amoxicillin, and metronidazole) twice a day for a week; turmeric tablets (700 mg) three times a day for 28 days.	Human	*H. pylori* patients were randomized in two groups: triple therapy group (*n* = 50); and triple therapy + curcumin group (*n* = 50).	Significantly decreased malondialdehyde markers, glutathione peroxides and increased total antioxidant capacity of the gastric mucosa in standard triple with curcumin group. The eradication rate was significantly increased in triple therapy + curcumin treated patients.	(47)
Curcumin	Curcumin 500 mg / day in combination with standard triple therapy (clarithromycin 500 mg, amoxicillin 1,000 mg, and pantoprazole 40 mg twice daily)	Human	68 *H. pylori* infected patients with peptic ulcer were divided into two groups: curcumin group (curcumin + standard triple therapy) (*n* = 33); placebo group (standard triple therapy) (*n* = 35).	Significantly improved dyspepsia in curcumin group. The eradication rate of *H. pylori* is equal in both groups.	(48)
Curcumin	Curcumin 30 mg, bovine lactoferrin 100 mg, N-acetylcysteine 600 mg, and pantoprazole 20 mg twice daily for a week.	Human	25 *H.pylori* positive patients with functional dyspepsia	Significantly improved functional dyspeptic symptoms and serologic signs of gastric inflammation in all patients, and the eradication rate of *H. pylori* was 12%.	(49)
Curcumin	A four-week course of turmeric tablet (700 mg orally three times a day).	Human	36 *H. pylori* infected patients were randomly assigned to receive curcumin (*n* = 17) or Omeprazole-based triple therapy regimen (n = 19) including Amoxicillin, Metronidazole and Omeprazole.	The eradication rate of *H. pylori* in single curcumin group (5.9%) far lower than conventional triple therapy (78.9%). No inhibition of IL-8 mRNA expression was observed in curcumin group.	(50)
Garlic	Water-ethanol extract	Mongolian gerbils	Garlic extract was fed to *H. pylori*-infected or *H.pylor*i-free Mongolian gerbils in their diets at doses of 1, 2 and 4% from 4 h to 6 weeks after inoculation, or at a dose of 4% from 2 to 6 weeks after inoculation.	Garlic extract could suppressed *H. pylori*-induced gastritis in a dose-dependent manner, especially at 4% concentration. However, the number of viable *H. pylori* was not affected by the garlic extract.	(53)
Garlic	Garlic powder 4 g daily for 8 weeks	Human being	36 patients were divided two groups: treatment group with mean years of 40.87 ± 16.45 received 4 g daily garlic powder, omeprazole 500 mg, amoxicillin 1 g, Bismuth 1.5 g and metronidazole 500 mg; control group (35.40 ± 11.26 years) only received the same antibiotics.	87% *H. pylori* negative cases in garlic treated group and 73% *H. pylori* negative cases in placebo group, which tested and evaluated by UBT. However, the different was not significantly.	(54)

### Suppressive effects of edible herbs on *Helicobacter pylori* infection *in vivo*

China have rich experience in using medicinal herbs to manage stomach diseases over the long history, which is gaining popularity recently. Parts of medicinal herbs were developed as food because of their safe and functional properties.

Licorice root (*Glycyrrhiza glabra L.*), which could be found in almost all Traditional Chinese Medicine (TCM) regimens. It is a common practice to use *G. glabra* to treat gastric ulcers in TCM. Aqueous licorice root extract had shown anti-adhesion effect on *H. pylori* by interfering with the binding action between the bacterial adhesins and human gastric tissue (55). Administration of *G. glabra* extract had anti-*H. pylori* effect in both C57B/L mice and Mongolian gerbils (56).

Licorice root extract showed a significant reduction of *H. pylori* load compared to the placebo group. The results also showed that the eradication of *H. pylori* by stool antigen test in GutGuard treatment group was 56% compared to 4% in the placebo group (57). Adding licorice to the clarithromycin-based triple therapy could significantly increase the *H. pylori* eradication rate reached 83.3% over the control group (62.5%) (58). Moreover, fermented milk in combination with licorice could significantly reduce *H. pylori* density and relieve gastrointestinal symptoms and histological inflammation (59).

*Hericium erinaceus* referred to as the lion’s mane mushroom or monkey’s head mushroom (in Chinese), can be used as both edible and medicinal fungus. *H. erinaceus* has been widely used in TCM to treat chronic superficial gastritis and gastric ulcers (60). Wang et al. (60) showed that the ethanolic extracts of *H. erinaceus* significantly reduced the *H. pylori* colonization in the stomach of mice (60). Despite the fact that monkey head mushrooms have been used in Chinese medicine for thousands of years, however, few clinical studies have examined the suppressive effects of *H. erinaceus* on *H. pylori* infection. In addition, *Tremella mesenterica* is another fungus found to have anti-*H. pylori* activity. Clinical study showed that *T. mesenterica* had immunomodulatory effect on *H. pylori-*infected patients when administered 2 g/day for 10 days (61).

Green tea is one of the most popular beverages in China and Japan. Green tea has been found to have suppressive effects on *H. pylori* growth (62). Intake of green tea in advance could attenuate this microbe colonization and gastric mucosal inflammation before infection (62). Matsubara et al. had found that green tea aqueous extract exerted a dose-dependent suppressive effect under three different concentration (500, 1,000 and 2000 ppm) on gastritis and the prevalence of *H. pylori* in infected Mongolian gerbils (63). One of main antioxidant compounds in green tea was catechins, which showed antibacterial activity against *H. pylori in vivo* (51). In infected Mongolian gerbils, *H. pylori* was eradicated in 36% animals with the dietary intake of 2% catechins for 14 days, and with significant decreases mucosal hemorrhage and erosion (64). The authors indicated that tea catechins have an anti-*H. pylori* effect and may have a therapeutic effect against gastic mucosa injury induced by *H. pylori*.

Taken together, edible herbs share in nature the characteristics of food and medicine. Some common but important edible herbs with anti-*H. pylori* effect including licorice, fungus and green tea were selected for this review (Table 4). All results have shown that edible herbs inhibit *H. pylori* colonization to some extend and improve *H. pylori*-related symptoms, likely by attenuating the development of inflammation. It is worth noting that numerous edible herbs are an invaluable treasure for the Chinese and people of East Asia. The potential of edible herbs to combat gastric disorders, particularly *Helicobacter pylori* infection, is worthy to be explored in the future.

**Table 4:** Suppressive effects of edible herbs on *Helicobacter pylori* infection *in vivo.*

Edible herbs	Components	*In vivo* models	Study sample	Main results	References
Liquorice	The extract of root of *Glycyrrhiza glabra* (Trade name: GutGard)	Mongolian gerbils and C57BL/6 mouse models	Infected mouse were orally treated once daily 6 weeks / week for 8 weeks with 15, 30 and 60 mg/kg of the extract.	25 mg/kg extract of *G. glabra* significantly reduced *H. pylori* colonization both in animals.	(56)
Liquorice	The extract of root of *G. glabra,* 150 mg once daily for 60 days. (Trade name: GutGard)	Human with *H. pylori* infection	107 patients with *H. pylori* infection aged 18–45 years. Patients who were divided into two groups received 150 mg GutGard (*n* = 55) or placebo group (*n* = 50), respectively.	Significantly decrease in the *H. pylori* load as compared to the placebo group. The negative rate of *H. pylori* Stool Antigen test was 56% in treatment group as compared to placebo group (only 4%).	(57)
Liquorice	380 mg *G. glabra* adding to Clarithromycin-based triple therapy, twice per day for 14 days.	Human with *H. pylori* suffering from non-ulcer dyspepsia or peptic ulcer	120 patients with non-ulcer dyspepsia or peptic ulcer disease randomized into treatment group (Clarithromycin-based triple therapy + *G. glabra*) and control group (Clarithromycin-based triple therapy only).	The eradication rate of *H. pylori* was 83.3 and 62.5%, respectively.	(58)
Liquorice	Fermented milk containing 100 mg *G. glabra*	Human with *H. pylori* infection aged 19–70	142 patients were randomly assigned to the treatment group (*n* = 71) or placebo group (*n* = 71). Treatment group received fermented milk with *G. glabra* and placebo group received only fermented milk.	The value of ^13^C urea breath test at 8 weeks was significantly reduced in the treatment group. Chronic inflammation, gastrointestinal symptoms and quality of life improved significantly in the treatment group.	(59)
*H.erinaceus*	Ethanolic extracts	C57BL mice	Mice infected *H. pylori* were divided into two groups including control (no extract) and *H.erinaceus* extract group (50 mg / mouse / day), following feeding for 20 days.	*H.erinaceus* extracts can attenuate the *H. pylori* load in the stomach of mouse.	(60)
*Tremella mesenterica*	T Submerged cultivated *mesenterica mycelium*	Human being	Fifty-two patients infected *H. pylori* were assigned three groups: group A (omeprazole 20 mg, amoxicillin 1,000 mg and clarithromycin 500 mg); Group B (*T. mesenterica* 2 g + omeprazole 20 mg); Group C (*T. mesenterica* 2 g only); Three groups were administrated for 10 days.	The treatment with *T. mesenterica* was not much effective on eradicating *H. pylori*. However, fewer adverse effects and a significant symptomatic relief in treated patients.	(61)
Green tea	Aqueous extract of green tea with a concentration of 1%.	C57BL/6 J	1 20 mice was randomly divided into four groups: Control (*n* = 5): water; Green tea group (*n* = 5): green tea; Green tea / infection / Green tea group: green tea for 2 weeks before infection and then green tea for 6 weeks; Infection / Green tea group: green tea for 8 weeks immediately after infection.	Green tea consumption could decrease the number of *H. pylori* and prevent gastric mucosal inflammation when mice ingested green tea prior to infection.	(62)
Green tea	Green tea extract	Mongolian gerbils	*H.pylori* (+) groups received green tea supplement (0, 500, 1,000 or 2000 ppm); *H.pylori* (−) groups: received green tea supplement (0, or 2000 ppm);	Infected Mongolian gerbils drunk green tea could suppress gastritis and the prevalence of *H. pylori* in a dose-dependent manner.	(63)
Green tea	Catechins	Mongolian gerbils	Gerbils were randomly divided into four groups after bacterial inoculation. Four weeks after bacterial inoculation, each group of gerbils was fed different diet containing 0, 0.5, 1 or 2% catechins for 2 weeks, respectively.	Decreased the number of H.pylori in the stomoch and significantly prevented gastritis	(64)

## Conclusion

Taken together, the results in this review are not completely satisfactory because in most cases *H. pylori* eradication is not obtained. However, this review showed that *H. pylori* colonization, severity of gastrointestinal inflammation and antibiotic therapy results would be improved by dietary ingredients from natural food resources. From the human beings perspective, the alternative treatment by single or combination of dietary ingredients from natural food resources to reduce *H. pylori* colonization is a promising and valuable strategy. The underlined mechanism of action has not yet been well explored about these natural food resources. Meanwhile, the safety and efficacy of the active compounds isolated from these natural foods are also lack. In future, it is necessary to further formulate active ingredients and make them available for clinical use.

Given the fact that complete eradication with natural foods or dietary ingredients may be impractical. The supplement of these foodstuff may provide a useful adjunct or alternative to conventional drug treatments, and should be tested in infected human for clinical trials. The natural products and dietary ingredients might even shift the consumers’ life patterns due to their low cost and relative safety after consuming for a long time. Moreover, the decline of excessive antibiotic use would have profound effects on human health and environment, including reduction of antibiotic resistance and protection of human microbiota diversity.

## Author contributions

CW: Writing – original draft. MY: Writing – review & editing. HZ: Writing – review & editing. SM: Writing – review & editing. FS: Writing – review & editing. SN: Writing – review & editing. MX: Writing – review & editing.
